# 

**Published:** 1915-08-14

**Authors:** 


### Medical Appointments.

Dr. Mungaldas T. Khandwalla has been appointed junior assistant medical superintendent at the St. Pancras North Infirmary, Highgate.

Dr. Nusserwanjee S. Paruck, M.B., B.S. (Bombay), junior assistant medical superintendent at the St. Pancras North Infirmary, Highgate, has been promoted to the post of senior assistant medical superintendent rendered vacant by the resignation of Dr. White.

HONOUR FOR A SENIOR PHYSICIAN.

Dr. H. Johnstone Campbell, the senior honorary physician to the Bradford Royal Infirmary, has completed twenty years in that post, and has received the cordial thanks of the governors for the valuable services he has rendered to the institution. The board of management has resolved that he be reappointed honorary physician for a further period of five years. Dr. Campbell is an M.D. and F.R.C.P. Lond., and is Professor of Therapeutics and Pharmacology in the University of Leeds. He is a consulting physician to the Bradford Royal Eye and Ear Hospital, the Victoria Hospital, Keighley, and the Bingley Cottage Hospital.

### The Freund Bequest to Addenbrooke's Hospital.

Miss Ida Freund, of Newnham College, who died early in 1914, bequeathed the sum of £2,000, duty free, to Addenbrooke's Hospital, Cambridge. As the major part of the deceased lady's estate consisted of foreign securities the executors of the will feared that they would not be able to realise the stocks, and in consequence would be unable to pay the bequest to Addenbrooke's for an indefinite period. However, the General Committee have received recently from the executors a cheque for £1,200 on account, and they hope to be in a position to pay the balance at a future date. In the case of Miss Ida Freund's bequest a bed is being named the "Ida Freund Bed" to perpetuate her memory, and a brass engraved tablet will be erected over same, in addition to one of the executors of the will being appointed an honorary governor for life.

### Latest Installations in Hospital Equipment.

### WHAT OTHERS ARE DOING.

*** **We desire information calculated to keep this column up to date in the interest of institutional workers generally. Secretaries and other executive officers are invitcd to forward particulars of new installations in any department. In this way a file will be available for all hospital authorities contemplating improvements and anxious to know what other institutions have found effective and moderr.**

The Empire Hospital, Westminster, has installed a system of light signals in place of the electric-bell service. The work has been carried out by Messrs. H. J. Cash and Co., Ltd., of Caxton House, Westminster, at a cost of £85.

The Empire Hospital, Westminster, as to the whole of the interior, has been redecorated in Duresco with a Silpako finish. The work has been carried out by Messrs. G. J. Chapple and Son, of Vauxhall Bridge Road, S.W., at a cost of about £300.

### The Book Market.

### Pamphlets and Papers.

What Shall we Drink? By John Kyte Collett, Cardiff. Price 1d.

How Belgium is Fed. Being extracts from an article in the *North-Western Review*. By William C. Edgar. Published by the National Committee for the Relief of Belgium.

**Rates of Subscription:** Twelve months, 6/6; Six months, 4/-; Three months, 2/-, post free to any address in the United Kingdom. Abroad, Twelve months, 8/8; Six months, 5/-; Three months, 2/6, post free.—Address The Subscription Dept., "The Hospital," The Hospital Building, 28/29 Southampton Street, Strand, London, W.C.

